# Biomaterializing the advances in uterine tissue engineering

**DOI:** 10.1016/j.isci.2022.105657

**Published:** 2022-11-24

**Authors:** Zhangming Wei, Yi Hu, Xiang He, Wen Ling, Jinxin Yao, Zhenjuan Li, Qiru Wang, Liping Li

**Affiliations:** 1 Department of Obstetrics, The Second Clinical Medical College, Jinan University (Shenzhen People’s Hospital), Shenzhen 518020, China; 2 The First Affiliated Hospital, Jinan University, Guangzhou 510000, China; 3 The Second Xiangya Hospital, Central South University, Changsha, Hunan 410000, China; 4 The First Affiliated Hospital, Department of Obstetrics and Gynaecology, Hengyang Medical School, University of South China, Hengyang, Hunan 421001, China

**Keywords:** biological sciences, bioengineering, tissue engineering, biotechnology

## Abstract

The uterus is considered to be a unique wound-healing model and distinguished by the repeated shedding of the endometrium and self-traceless regeneration. Common curettage, cesarean section, and other operations often cause endometrial and myometrial defects and obstetric and gynecological complications, leading to a high demand for uterine repair or partial replacement. However, the structure and function of the uterus are complicated. Functional uterine tissue engineering requires highly specialized biomaterials with a natural extracellular microenvironment. Currently, no biomaterial can fully simulate the structural and biomechanical properties of the uterus. Many efforts have been made to develop highly functional materials and tissue structures that may provide uterine tissue engineering constructs for reducing obstetric and gynecological complications. Continuous efforts will likely facilitate the development of scalable cells and biomaterial technologies for clinical use. This review summarizes the recent applications of biomaterials and tissue engineering in rebuilding a portion of or the entire uterus.

## Graphical abstract

**Figure:**
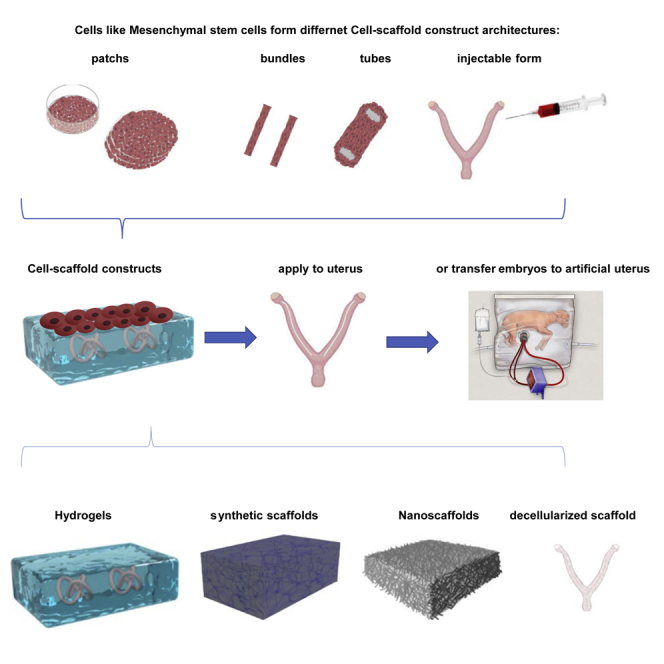

## Introduction

The uterus is a unique wound-healing model. According to research, during menstruation, the uterus may undergo scarless repair via inflammatory responses, proliferation, intrinsic endometrial stem cell (EndoSC) differentiation, and tissue remodeling.^1^ Various pathological factors, such as uterine operation, curettage, uterine ischemia, and infection, cause uterine injuries such as intrauterine adhesions (IUA), and 210 million women become pregnant and 140 million infants are born each year; approximately two-thirds to one-third of women require cesarean section.^2^ More than 80% of patients had a series of high-risk obstetric complications as a result of cesarean section wounds and defects.^3^^,^^4^

**Figure 1:**
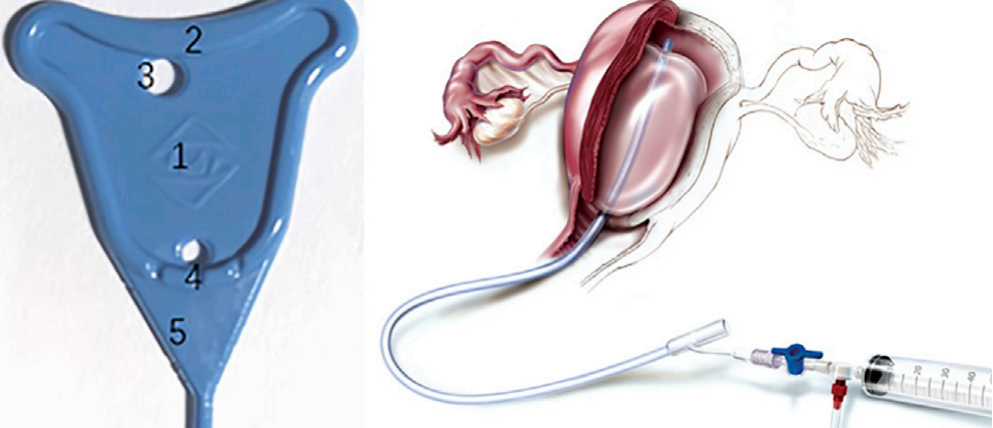
Rubber stents and intrauterine balloons currently in clinical use for IUA

Intrauterine devices, such as metal or rubber stents and intrauterine balloons, are currently used in clinical settings after hysteroscopic adhesion lysis (Figure 1).^5^ Although every study reported positive results, existing treatment methods did not reduce the majority of complications.^6^ This has gained prominence, particularly because of young people’s fear of childbearing,^7^ the year-by-year decline in fertility rates,^8^ and changes in fertility policies, such as China’s Third Child Policy.^9^

Artificial bioactive substances are used in uterine tissue engineering to repair or build organs and tissues.^10^ Biology, materials science, engineering, and other disciplines are all involved.^11^ Artificial uteri and tissue-engineered uteri have become a reality in the 21st century. Although the use of an artificial uterus for complete childbearing is still not widely explored, research into uterine tissue engineering has recently been developed.^12^ However, current challenges in tissue engineering include cultivating more complex tissues, effectively facilitating tissue regeneration, and improving the biomechanical stability of transplanted constructs.^13^ The uterus is composed of the serosal myometrial and endometrial layers. The endometrial layer consists of three layers: functional, basal, and compact. The functional layer performs an endocrine function, which is influenced by hormones, to support the pregnant fetus and the endocrine system. Scaffolds with adjustable variable physical-mechanical properties in uterine tissue engineering can replace the uterine myometrial serosal layer for deformation maintenance during pregnancy. Electrospinning, for example, can mimic the structure of multilayered myofiber bundles in the uterus, and the regenerative capacity of the human endometrium requires a population of local stem cells. Transplanted endometrial cells offer the highest efficiency for secreting and nutritionally supporting fetal development, and uterine tissue engineering is the optimal solution for treating uterine defects. Tissue engineering consists of three components: cells, cell-scaffold construct architectures, and scaffold materials.

### Cells used in uterine tissue engineering

The advancement of cells and tissue engineering technology provides treatment options for uterine reproductive function remodeling.

### Mesenchymal stem cells (MSCs)

MSCs are important in regenerative medicine.^14^ MSCs are suitable for tissue engineering applications due to four characteristics: their ability to home to the injured site, the ability to differentiate into various cells, the secretion of bioactive molecules, and immunomodulatory functions.^15^

### Bone marrow-derived MSCs (BMSCs)

BMSCs are derived from the bone marrow and offer limited clinical application. Researchers have demonstrated that BMSCs play an important role in endometrial reconstitution,^16^^,^^17^^,^^18^ which cannot be accomplished directly through cell replacement.^19^^,^^20^ According to Ho et al., endometrial repair is likely related to the conditional medium of BMSCs.^21^ Autologous cells are collected from the patient as a source of BMSCs; such cell lines require effort and time to use because they must be cultured from the patient’s bone marrow.^22^ Autologous CD133^+^ BMSCs have been used in stage II/III clinical trials for uterine and endometrial repair with significant efficacy and safety.^23^

### Adipose-derived stem cells (ADSCs)

ADSCs can be obtained more easily and safely than BMSCs.^24^ Sun et al.^25^ demonstrated that ADSCs could be used to regenerate uterine horns in a rat model. Farhad et al.^26^ demonstrated that local injection of ADSCs, rather than BMSCs, is more appropriate for treating Asherman’s syndrome by removing fibrosis and replacing epithelial cells. Furthermore, ADSCs are likely to repair the damaged endometrium via secreted exosomes, which increase VEGF1 and insulin-like growth factors-1 (IGF1) expression and facilitate ADSC migration and recruitment.^27^

### Umbilical cord mesenchymal stem cells (UC-MSCs)

Human UC-MSCs isolated from discarded umbilical cord tissue offer numerous advantages, including a large source, convenient access, rapid self-renewal ability, low immunogenicity, and no ethical controversy. It is an exceptional cell source for the development of tissue-engineered products.^28^ In recent years, UC-MSCs have been widely used and have demonstrated positive regenerative effects in rat uterine injuries. Cao et al.^29^ performed a phase I clinical trial in which they seeded UC-MSCs into a collagen patch, rolled the collagen patch on a urinary catheter, extended it into the patient’s uterus, and then filled it with water to stretch the patches and adhere them to the uterine wall. This collagen membrane degrades, causing no harm to the human body. These findings demonstrate that after treatment endometrial proliferation and neovascularization improve. UC-MSCs could be used on a large scale in the near future.

### Embryonic stem cells

Human embryonic stem cells (hESCs) can proliferate and differentiate into cell types from all three germ layers indefinitely. Song et al.^30^ used an endometrial stromal cell co-culture system to induce hESC differentiation into endometrium-like cells and demonstrated uterine horn regeneration in rats. hESCs offer a high differentiation and proliferation potential, which should be investigated further in the repair and replacement of complex uterine tissue defects. Certain defects of hESCs may be responsible for tumorigenesis.

### Uterine stem cells

The uterus comprises myometrium and endometrium. Myometrial stem cells and EndoSCs are examples of uterine stem cells.^23^ The evidence of myometrial stem cells is less than that of EndoSCs. The endometrium plays an important role in the menstrual and reproductive processes in women. Myometrial stem cells have rarely been investigated in the field of uterine tissue engineering.^23^ During each menstrual cycle, a dynamic balance between endometrial impairment and repair must be maintained. Otherwise, a series of diseases and disorders such as uterine bleeding, infertility, miscarriage, pregnancy complications, endometriosis, and endometrial cancer may develop. EndoSCs reside in the basalis and are mostly destroyed and rebuilt in the functional layer. EndoSCs are progenitor cells that differentiate into the endometrium. They provide damaged functional layers to progenitors, ensuring a normal menstrual cycle. EndoSCs can also develop into cartilage,^31^ cardiomyocytes,^32^ adipose tissue^33^ and neurons.^34^

EndoSCs were used in early clinical trials to repair the uterine endometrium. EndoSCs have the potential to treat thin endometrium,^35^ IUA, and infertility. Canosa et al.^36^ have suggested that EndoSCs can differentiate into endothelial cells *in vitro* when co-cultured with human umbilical vein endothelial cells (HUVEC) and EndoSCs possess angiogenic properties. According to researchers, EndoSCs differ from other stem cells in terms of their therapeutic roles. Liu et al.^37^ compared the recovering effect on the endometrium of BMSCs and uterine-derived cells. Their results have suggested that endometrial-derived cells are less effective than BMSCs in regenerating the endometrium, and systemic administration of stem cells was discovered to be more effective than local injection. Their findings also revealed that direct local injection can result in significant cell loss; further studies can use biomaterials to deliver and support transplanted cells. Yin et al. [34] discovered that CD34^+^KLF4^+^ stromal-resident stem cells activate and integrate into the regeneration area, where they differentiate and integrate into the endometrial epithelium *in vitro*, and further *in vivo* therapeutic research is being considered.^38^ Han et al.^39^ discovered that human amnion-derived MSCs could be used in uterine stem cell therapies. Human amnion membrane cells elicit an immune response when transplanted into most human tissues, but they are similar to uterine mesenchymal cells in terms of cell markers and HOXA and WNT5a gene expression patterns. These similarities are not observed in MSCs derived from amniotic fluid or umbilical cord; however, their efficacy and potential further use have not been demonstrated in the literature. Endometrial stromal cells can proliferate and differentiate into functional endometrial layers. Endometrial stromal cells have also been used to repair the endometrium; however, nude mice were used as therapeutic receptors.^40^ Significant differences exist between immunodeficient nude mice and humans, including reproductive physiology, immunity, uterine anatomy, and long-term immunosuppressive agents. Furthermore, collecting and proliferating endometrial cells from endometrial disease patients for transplantation is challenging. Obtaining a large number of endometrial cells is also difficult, particularly in the endometrial epithelium.^41^ Li et al. isolated human endometrial perivascular cells (CD146^+^PDGFRβ^+^) that function as EndoSCss and discovered that the overexpression-CYR61 endometrial perivascular cell group exhibited significantly higher blood vessel distribution.^42^ Modified human endometrial perivascular cells are expected to be investigated further in endometrial tissue regeneration.

### Oral epithelial cells

Some researchers have used oral mucosal epithelial cell sheets to prevent IUA and subsequent infertility. This reduces the likelihood of readhesion^43^ and can prevent fibrosis and maintain uterine cavity morphology. However, the effects of regeneration are unknown.

### Cell-scaffold construct architectures

**Figure 2:**
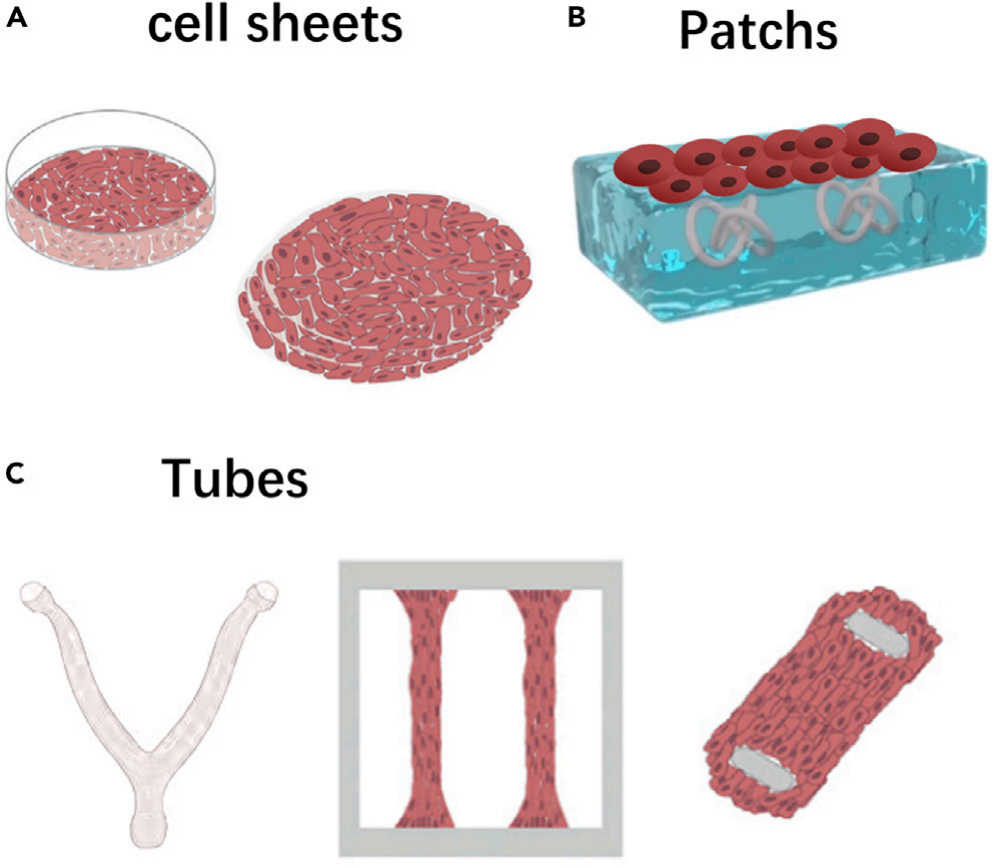
Cell construction in uterine tissue engineering (A) Cell sheets; (B) Patchs; and (C) Tubes.

Applications of uterine tissue engineering technology are expected to grow rapidly over the next decade. The production of various tissue structures that can be used for specific engineering and scientific purposes is part of the appeal of uterine tissue engineering strategies. Tissue engineering strategies can be used to develop various organizational structures for specific engineering and scientific purposes. Among these tissue structures are stackable sheets of cells, tissue patches with few cells, and large cylindrical structures of clinically relevant uterine-like organs. We have described some of the most common organizational structures used by various organizations (Figure 2).

### Sheets

The cell sheet technique has been used in several studies with oral mucosal epithelial cells, endometrial stromal cells, and endometrial epithelial cells.^25^ This structure has the advantage of being highly biocompatible. The disadvantages include a lack of cell support and elaborate structure, a fragile physical structure, a low cell survival rate, low adhesion, a rapid metabolic degradation rate, and a high cell requirement.

### Patches

More uterine tissue engineering strategies are in this form and have advanced to the clinical trial stage. Cao et al.^29^ used UC-MSCs and a collagen patch to treat human uterine injuries, as previously mentioned. Other patches contain various biomaterial bases and cells.^44^^,^^45^^,^^46^^,^^47^ Some researchers believe that tissues can only grow in pores larger than 100 μm in diameter and that pore size and internal connectivity also influence tissue growth type. Polycaprolactone (PCL) electrospun scaffolds can precisely adjust the pore size of scaffolds to the nanoscale.^48^ The structure of multilayered myofiber bundles in the uterus is mimicked by electrospinning. It has shown promising results when seeded with EndoSCs to promote tissue regeneration in full-thickness skin wounds.^49^ Electrospinning patches can be investigated for uterine tissue engineering patch formation in the near future.

### The shape of the injury (injectable)

The injectable hydrogel has given gynecological patients hope. It reduces abnormal mechanical loading caused by tissue bulking and improves adaptive remodeling caused by inflammatory changes, but clinical trial results are not consistent in diseases such as myocardial infarction. The factors influencing hydrogel degradation in the damaged area following injection are unknown, resulting in varying stem cell and drug viabilities in the body.^50^ In IUA, several injectable hydrogels exhibit promising anti-fibrosis effects.^51^^,^^52^^,^^53^ Therefore, we need to determine the optimal proportion of injectable hydrogel components to treat uterine injuries. To overcome the aforementioned challenges, we need to develop a noninvasive, highly effective, and quick injection route. Injectable hydrogels and gene therapy can be used in conjunction to target the microenvironment, extend the viability of cells and drugs in the damaged area of the uterus, and achieve cell regeneration to repair the injured or damaged uterus.^50^

### Tubes

Several studies have demonstrated the synthesis of engineered tissues that resemble natural uterine horns. To substitute a severely traumatic uterus, they designed a decellularized scaffold and recellularized it to form a tube shape, a uterine horn-like organoid, or the entire uterus. Maruyama et al.^54^ decellularized the uterus to obtain an extracellular matrix (ECM) scaffold before injecting primary uterine cells and MSCs from another mouse into the scaffold wall. Endometrioid-like tissue was observed following 3 days of perfusion culture, but there was no robust smooth muscle layer. In the excision and cell scaffold replacement groups, the pregnancy rate was 75%, which was comparable to that of the normal functional uterus. This structure could be used for high-throughput drug testing, uterine replacement, and other applications.^55^

### Scaffold materials

**Figure 3:**
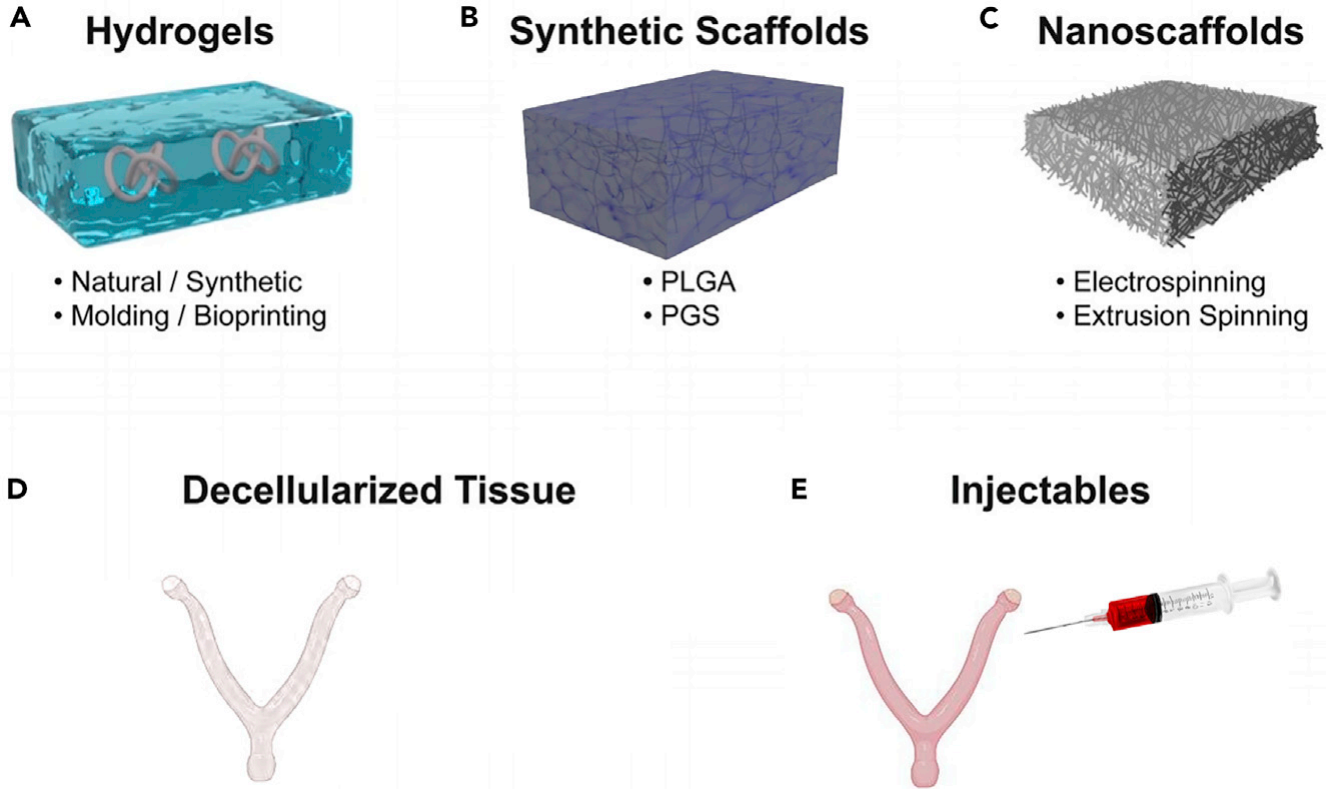
Biomaterials and techniques in uterine tissue engineering (A) Hydrogels; (B) Synthetic scaffolds; (C) Nanoscaffolds; (D) Decellularized tissues; (E) Injectables.

Endometrium biomaterials must have optimized pore size, porosity, mechanical properties, composition, cell adhesion, and compatibility.^56^ Hydrogels are ideal scaffolds for carrying stem cells due to their high water content, suitable microenvironment, and adjustable mechanical properties.^42^ Bai et al. demonstrated that the hydrogel structure could cause encapsulated MSCs to spontaneously differentiate. Hydrogel’s stiffness,^57^ degradation,^58^ and viscoelastic properties^59^ all influence cell differentiation and secretion. Yang et al.^60^ investigated the effect of hydrogel stiffness on MSCs using dynamic softening hydrogels and discovered that MSCs exhibited mechanical memory and retained their differentiation ability even when the matrix hydrogels were softened. The degradation of hydrogels can influence the diffusion of water-soluble factors, affecting tissue recovery.^58^ Other research has discovered that degradability is beneficial for maintaining human myeloid nuclear stem cells.^61^ Some hydrogels are degraded by stem cell-secreted factors or send signals to stem cells via by-products.^62^ To simulate an anoxic environment and regulate the secretion of stem cell-related factors, hydrogels can be easily modified by releasing dimethyloxalylglycine.^63^ To treat IUA, many hydrogels and scaffolds have been developed, which work with cells to promote endometrial regeneration (Figure 3, Table 1).^64^

**Table 1:** Biomaterial substrates used for uterine tissue engineering

Scaffold material	Advantages	Disadvantages	Source	Cell loaded	Architectures
Collagen	Native uterine ECM protein; adhesiveness	Poor mechanical properties	Nature-derived	BMSCs; UC-MSCs	Sheets; Patch; Injectable
PF-127	Fast gelation; scalable	Cell toxicity	Synthetic	BMSCs	Patch; Injectable
Alginate	Biocompatibility; easy drug-load; porous	High-inflammation-response, Low- tenacity	Nature-derived	–	Patch
Chitosan	Biocompatibility, antibacterial activity	Difficulty to remodel; poor viscosity	Nature-derived	BMSCs; UC-MSCs, Endometrial stromal cell	Patch
Hyaluronic Acid (HA)	Readily modifiable; clinically approved; scalable	Difficulty to remodel; poor viscosity	Nature-derived / Synthetic	–	Patch
Gelatin	Low antigenicity	Rapid degradation	Nature-derived	EndoEpCS and EndoSCs	Patch;
Poly(Lactic-co-glycolic) acid(PLGA)	High porosity; clinically approved	High stiffness	Synthetic	BMSCs	Patch
Polyglycerol sebacate (PGS)	Viscosity; cheap	Fatigability; low porosity; long degradation time	Synthetic	BMSCs	Patch;
Rabbit Amniotic membrane decellularized	Native uterine ECM; high biocompatibility	Xenogenicity; scalability; reproducibility	Nature- derived	–	Patch; Tube
Human Amniotic membrane decellularized	Native uterine ECM; Scalable	Reproducibility	Nature-derived	–	Patch; Tube
Rodent uterus Decellularized scaffold	Native uterine ECM; high biocompatibility	Xenogenicity; scalability; reproducibility	Nature-derived	human myocytes; rodent myocytes	Tube; Whole uterus
Porcine uterus Decellularized scaffold	Native uterine ECM; human-scale tissue;	Xenogenicity; scalability; reproducibility	Nature-derived	human side population stem cells	Tube; Whole uterus

### Collagen

Collagen is a protein hydrogel that forms an important part of the ECM. It contains arg–gly–asp (RGD) crosslinking sites that aid in cell attachment.^65^ We can simulate the function of the target tissue and provide the critical factors needed to regulate dynamic cell behavior and intercellular communication^66^ by selecting the appropriate collagen and assembly process.^67^ It can support cell adhesion, differentiation, and migration as a scaffold for exogenous cells.

Collagen viability is high in stem cells used in the uterus, such as BMSCs and UC-MSC. Hong^68^ used rabbit myometrium, endometrium, and epithelial cells in a collagen/matrix mixture to form an *in vitro* uterine wall-like layered structure to support mouse embryo development. In a rat uterine injury model, Xu et al.^47^ investigated the role of collagen/UC-MSCs in promoting collagen degradation. UC-MSCs were discovered to reduce collagen deposition in the uterine scar, increase pregnancy rates, and repair the structure by secreting matrix metalloproteinase (MMP-9). In terms of vascular density, structural repair, and fetal implantation, Li^42^ discovered that the collagen/En-PSC overexpressing CYR61 (cysteine-rich angiogenesis inducer 61) group outperformed the other groups. The mechanical stiffness of common collagen scaffolds, however, is insufficient.^67^ They undergo fibroblast-mediated contraction following *in vivo* implantation, reducing their adhesion ability and absorbing quickly.^69^ Stratesteffen et al.^70^ developed a photo-crosslinking of collagen and methacrylate gelatin that provides attachment sites for HUVEC and hMSC, promotes angiogenesis, and may have applications in IUA treatment.^44^^,^^45^ Researchers developed collagen delivery systems by combining collagen membranes with leukemia inhibitory factor,^71^ vascular endothelial growth factor,^72^ and fibroblast growth factor ,^73^ which were then transplanted into the rat uterus in a uterine full-thickness injury rat model, and then compared the experimental group construction and fertility function restoration to the other control groups. The results demonstrated that the collagen scaffold with cytokines increased the endometrial thickness, and the embryos were implanted successfully.

### Gelatin

Gelatin is primarily composed of natural collagen that has been deformed or partially hydrolyzed. Gelatin is less antigenic than collagen because it lacks aromatic free radicals.^74^ Gelatin retains bioactive collagen sequences for cell attachment, such as RGD and MMP-9 degradation sites;^75^ gelatin can maintain growth factor biological activity through affinity fixation.^76^

At body temperature, gelatin is unstable and exhibits poor mechanical properties. Methylacrylamide gelatin (GelMA) can compensate for these shortcomings through photo-crosslinking to mimic endometrial tissue performance.^77^ Pence et al.^78^ encapsulated uterine epithelial and stromal cells (EndoEpCs and EndoSCs) in GelMA and demonstrated that EndoSCs and EndoEpCs improved angiogenesis. Zambuto et al.^77^ wrapped the above cells using GelMA, studied endometrial vessel formation, decidua reaction, and diffusivity from 2D and 3D perspectives, and detailed the critical characteristics of the above two cells in a 3D gelatin hydrogel.

### Alginate

To improve the mechanical properties of alginate gels, alginate, a low-strength, anionic natural polysaccharide polymer, can be electrostatically crosslinked with Ca^2+^ or chitosan, polylysine, and other polycations.^79^ Endometrial tissue recovery and uterine cell regeneration are aided by 3D-printed porous sodium alginate hydrogels loaded with human-induced pluripotent stem cell-derived MSCs.^80^

### Hyaluronic acid

Hyaluronic acid (HA) is a disaccharide-based glycosaminoglycan (d-glucuronic acid and N-acetylglucosamine). It is discovered in various connective, epithelial, and nervous tissues. Because of its exceptional hydrophilicity and biocompatibility, HA is widely used in biomedical materials. HA binds to the CD44 receptor and aids in embryo implantation.^81^ Furthermore, HA gel has also been used for the clinical prevention of IUA.^82^ According to Zhu et al., high molecular weight HA (1.2 × 10^6^ kd) is more conducive to reducing the degree of fibrosis after endometrial injury and preventing the formation of IUAs.^83^ Physiologically, HA-loaded stem cells offer a high potential for treating endometrial injuries. Kim et al.^40^ embedded endometrial stromal cells and different doses of fibrinogen and thrombin to treat IUA in mice. In rhesus monkeys, Wang^84^ loaded HUMSCs onto HA hydrogels to repair the endometrium. This complex exhibits strong anti-adhesion properties and promotes endometrial wall regeneration.

In terms of physical and chemical properties, HA also has the disadvantages of poor mechanical properties and low adhesion. The use of modified HA in endometrial clinical research may have a brighter future.^85^ Shin et al.^86^ developed a high-mechanical-strength hydroxy aldehyde crosslinking system for HA and aldehydes. Bermejo et al. developed an injectable hydrogel crosslinked with HA and cholic acid that has obvious protein adhesion and can prevent uncontrolled degradation.^87^ Guo et al. investigated the different crosslinking networks of HA and dopamine and their responses to pH.^88^ This network is notable for its remarkable adhesion and ability to tune the balance of dopamine and dopaquinone via mercaptan.

### Chitosan

Chitosan is a biocompatible and biodegradable polysaccharide extracted from chitin with antibacterial activity.^89^ Chitosan is a multi-cation molecule that can interact with negatively charged biomolecules due to amino protonation.^90^ Chitosan can also form electrostatic physical crosslinks with heparin and polyacrylic acid. To treat IUA, Qi et al.^91^ developed an stromal cell-derived factor (SDF)-1α controlled-release system by crosslinking chitosan with heparin. Chitosan has also been developed for use in the treatment of endometriosis using pigment epithelium-derived factor^92^ and small interfering RNA delivery systems.^84^ Chitosan exhibits the potential to be developed as a scaffold for encapsulating stem cells in uterine tissue engineering.

### Decellularized scaffolds

The ECM of human tissue provides mechanical support for cells and regulates cell proliferation and migration. 3D scaffolds developed by decellularization exhibit a promising future in tissue engineering and mimicking the internal environment. Santoso et al. investigated the efficacy and viability of decellularized rat uterine horns *in vivo*.^93^ Decellularized uterine tissue grafts were integrated within 30 days, demonstrating greater structural integrity than previously used scaffold materials, such as intestinal submucosa grafts.^94^ Another finding was that while SDS-decellularized tissues regenerated thicker and faster, high hydrostatic pressure samples preserved ECM content better due to less protein denaturation and no residual Triton X-100 or SDS. Furthermore, a lack of microvascular connections causes poor cell migration toward the graft, resulting in nutrient deficiency; decellularization of entire organs can be used to overcome this vasculature deficit.^93^ Hellstrom et al. discovered that a freeze-thaw protocol combined with repeated perfusion cycles with Triton X-100 and dimethyl sulfoxide (DMSO) produced the best results. Major histocompatibility complex classes I and II were also successfully removed.^95^ Another study^55^ discovered that using Triton X-100 and DMSO together resulted in better construction and restoration of fertility function in the uterine horn damage model.

Maruyama et al. decellularized uterine cells using a perfusion system containing SDS and Triton X-100 and then examined the decellularized scaffolds for their potential for recellularization *in vitro* and regeneration *in vivo*. To conduct an *in vitro* study with primary uterine and MSCs, the uterine wall was infiltrated. In the perfusion culture following 3 days, a smooth muscle layer and endometrial-like tissue were observed. To demonstrate regenerative ability, excision/replacement models were used, and the pregnancy rate was 75%, which is close to normal.^54^ Campo et al.^96^ used the repeat perfusion of Triton X-100 and DMSO to decellularize the mixture of stromal and epithelial side population stem cells from human endometrial recellularization results with/without the freeze/thawed uterus scaffold step in a pig model. They demonstrated that, regardless of freeze/thaw decellularization, these protocols can generate effective scaffolds in the pig uterus.

ECM components are difficult to characterize. Combining ECM-derived molecules with polymers, however, can affect specific cellular responses.^97^ Polyethylene glycol (PEG) is biocompatible and has tunable mechanical properties. It is simple to combine them with ECM-derived molecules by modifying their end groups. Victor et al.^98^ developed human intestinal enteroids and endometrial organoids from single cells using eight-arm PEG macromolecules grafted with integrin-binding peptides from fibronectin and collagen.

Miki et al.^99^ investigated how the orientation of decellularization scaffolds affected uterine tissue regeneration in a partially excised rat uterine model. They discovered that the uterus-placed reverse-orientation scaffolds exhibited a more atypical structure, while the pregnancy rates were comparable to those of the correct orientation groups. Yao^100^ compared the uterus reconstruction efficiency and best crosslinker concentrations of the naturally derived crosslinkers genipin (GP), procyanidins (PC), and glutaraldehyde (GA). They discovered that 90 days following transplantation, the decellularized extracellular matrix (dECM) scaffolds preserved the microstructure and vascular architecture in segmental circular uterine excision rats, while the crosslinkers GP and PC improved cell infiltration and caused low immune reactions, thereby promoting full-uterus repair. Decellularization scaffolds have some limitations as well. The exact composition of the ECM is difficult to determine, and residual cellular material remains a concern.

### Synthetic polymer scaffolds

Although natural polymers offer exceptional biocompatibility and adhesion properties, they are often expensive and have poor batch repeatability. During the separation process, microbial infections and potential immunogenicity must be avoided.^101^ Synthetic materials, in contrast, have the advantages of convenient manufacturing and tunable degradation, with some materials playing a specific role in molecule transfer.^76^ Endometrium synthetic materials should be nontoxic and attachable to cells.^101^

#### Pluronic F127

Pluronic F127 (PF127) is a triblock copolymer of hydrophilic poly (ethylene oxide) and hydrophobic poly-[propylene oxide], and at physiological temperature, PF127 gels at a liquid concentration of 15%–20% (w/w).^102^ PF127 is widely used to encapsulate cells and drugs because of this property.^103^ PF127 was discovered to protect against mitochondrial dysfunction,^104^ but it is highly toxic, with high cellular activity only at 0.1%–5% (w/w) concentrations.^102^ Therefore, PF127 is often combined with the antioxidant vitamin C (Vc) and the membrane stabilizer hydrocortisone to enhance cell viability, or low-concentration PF127 is crosslinked with alginic acid to enhance scaffold adhesiveness.^105^^,^^106^ Deng et al. investigated PF127-encapsulated BMSCs to collaborate with Vc in the treatment of endometrial damage based on this. Vc supplementation reduced inflammatory cytokines (IL-6 and TNF-α) and increased cell survival.^105^ PF127 loaded with sildenafil citrate, alginic acid, and hydroxyethylcellulose as adhesive agents increases endometrial thickness and uterine blood flow, potentially inducing pregnancy in patients.^106^

#### Poly- (glycerol sebacate)

Poly-(glycerol sebacate) (PGS) is a biocompatible polymer elastomer material with cell adhesion and degradation properties.^107^ PGS and BMSCs were studied and compared to collagen and poly-(lactic-co-glycolic acid) (PLGA) scaffolds in the treatment of IUA by Xiao et al.^56^ They discovered that PGS significantly increased BMSCs retention time and basic fibroblast growth factor (bFGF), vascular endothelial growth factor (VEGF), and transforming growth factor β (TGF-β) expression levels. Simultaneously, the endometrium recovered significantly. PGS, however, exhibits low mechanical strength and poor water absorption. The PGS can be modified to improve its performance and achieve the desired conditions.^107^ Fu^108^ used esterification to link palmitic acid to PGS as a vascular graft scaffold. This scaffold degrades more slowly than PGS, promoting the formation of arterial blood vessels and preventing bursts.

#### Poly (lactic-co-glycolic acid)

PLGA is a common drug carrier that transports dexamethasone^109^ and paclitaxel.^110^ Chen et al.^111^ used PLGA as a carrier for releasing estradiol on the human amniotic ECM scaffold to treat intrauterine adhesion. The findings revealed that PLGA exhibited no effect on the high porosity of the stent and was compatible with the uterus. PLGA can also be combined with PEG to develop a structure with tunable mechanical properties and degradability, improving drug release stability and having the potential to be used in the endometrium.^112^ Magalhaes used non-seeded PGA-PLGA scaffolds or autologous cell-seeded constructs to reconstruct the uterus after performing subtotal uterine excision in rabbits. Only the cell-seeded engineered uteri developed native tissue-like structures to support rabbit live birth six months postimplantation.^113^

#### Polycaprolactone

PCL is a polyester produced through the ring-opening polymerization of caprolactone monomers. It exhibits high biocompatibility and processability. However, PCL has poor mechanical properties, is hydrophilic, degrades slowly, and exhibits low cell affinity. It must be optimized for composites with other materials for further applications, and its mechanical properties can be improved by adjusting the physical structure of the fiber scaffold. Srividya compounded its surface with maltose, demonstrating that the synthetic material promoted uterine fibroblast proliferation.^114^^,^^115^ PCL is used as a biomaterial in electrospinning to develop nanofiber scaffolds that mimic the natural ECM. PCL nanofibers are highly hydrophobic, and the surface of PCL is modified by aminolysis, which introduces an amine group, which improves uterine cell attachment and proliferation.^115^

### Artificial uterus

**Figure 4:**
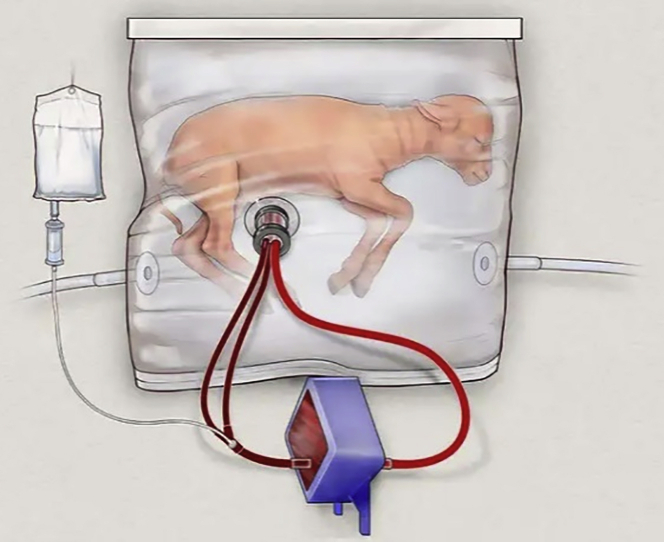
An extrauterine system to physiologically support the extreme premature lamb

A growing number of artificial uterine procedures are being developed. Emily,^116^ for example, developed an artificial extrauterine device to physiologically support an extremely premature goat infant for up to 4 weeks of gestational age. Most obstetricians believe that using synthetic devices throughout pregnancy is impossible, but these studies extend the time the systems can hold the fetus (Figure 4).

### Comparing other therapy options with tissue engineering

Current clinical treatments for uterine tissue defects are limited, and the majority of clinical outcomes are disappointing. The primary treatments used to restore the normal anatomical morphology of the uterine cavity are surgical adhesion loosening and estrogen. Following the operation, a contraceptive device, Foley balloon catheter, or uterine stent placed in the uterine cavity can aid in preventing the recurrence of uterine cavity adhesion and restoring morphology. The contraceptive device, however, cannot completely separate the anterior and posterior uterine walls, and adhesions may still occur. Simultaneously, it may cause local inflammation while using contraceptive devices, disrupting endometrial growth.^117^ Endometrial regeneration can be effectively promoted by covering the Foley balloon catheters with fresh amniotic membranes. This is a novel method of treating uterine cavity adhesions. However, nearly half of patients can develop adhesions after amniotic membrane transplantation,^118^ and the long-term efficacy of this treatment must be confirmed.

HA-assisted hysteroscopy to prevent adhesion recurrence is a therapeutic method that can form a membrane on the surface of the wound. However, there is no research conclusion on the pregnancy rate, which is currently only used for research purposes (Tables 2 and 3). According to the American Society of Gynecological Endoscopy guidelines, the use of contraceptive devices and Foley balloons after uterine cavity adhesion surgery is a potential infection risk factor; thus, using them outside of clinical trials is not recommended.^119^

**Table 2:** Recent clinical trials of biomaterial substrates for uterine tissue engineering

Trial name	Clinical trials.gov identifier	Substrate	cell	Estimated Enrollment	Uterine conditions
Treatment of Severe Asherman Syndrome by Collagen Scaffold Loaded With Autologous Bone Marrow Mononuclear Cells	NCT02680366	collagen scaffold	Autologous Bone Marrow Mononuclear Cells	144	Asherman Syndrome
Clinical Study of *in Situ* Regeneration of Endometrium	NCT04233892	Collagen Binding Domain-bFGF	Bone Marrow Mononuclear Cells	345	Infertility and Endometrial Fibrosis
Clinical Study of Umbilical Cord Mesenchymal Stem Cells Combined With Collagen Scaffold in the Treatment of Thin Endometrium	NCT03724617	collagen scaffold	umbilical cord mesenchymal stem cells	18	Thin Endometrium
The Efficacy and Safety of Collagen Scaffold Loaded With Umbilical Cord Derived Mesenchymal Stem Cells in Infertile Women With Thin Endometrium or Endometrial Scarring	NCT03592849	collagen scaffold	umbilical cord mesenchymal stem cells	50	Thin Endometrium or Endometrial Scarring
Treatment of Infertility by Collagen Scaffold Loaded With Autologous Bone Marrow Stem Cells	NCT02204358	collagen scaffold	autologous bone marrow stem cells	50	Infertility Intrauterine Adhesions Endometrial Dysplasia
Treatment of Infertility by Collagen Scaffold Loaded With Umbilical Cord Derived Mesenchyma Stem Cells	NCT02313415	collagen scaffold	umbilical cord mesenchymal stem cells	26	Infertility Intrauterine Adhesions

**Table 3:** Recent clinical trials of biomaterial used in uterine tissue engineering without cell

Trial name	Clinical trials.gov identifier	Substrate	Enrollment	Uterine conditions
Efficacy and Safety of Crosslinked Hyaluronan Gel for Preventing Intrauterine Adhesion	NCT02220621	Crosslinked hyaluronic acid gel	120	Intrauterine Adhesion
Seprafilm Slurry in the Prevention of Uterine Scarring in Patients Undergoing Hysteroscopic Myomectomy (Seprafilm)	NCT01632202	sodium hyaluronate (HA) and carboxymethylcellulose (CMC)	328	Hysteroscopic Myomectomy
HYALOBARRIER® GEL ENDO Versus+31:39 no HYALOBARRIER® GEL ENDO Following Operative Hysteroscopy for Improving Reproductive Outcome in Women With Intrauterine Pathology Wishing to Become Pregnant(AGNOHSTIC)	NCT03880435	Crosslinked hyaluronic acid gel	444	Infertility; Polyp Uterus; Myoma; Uterus Adhesion; Hysteroscopy; Uterine Septum; Retained Products of Conception
Intrauterine Adhesion Rate After Hysteroscopic Myomectomy and Application of HYALOBARRIER Gel	NCT01412489	Crosslinked hyaluronic acid gel	189	Myoma
Pivotal Clinical Study to Assess the Anti-adhesive Effect and Safety of ABT13107 Applied to Postoperative Intrauterine	NCT04007211	Crosslinked hyaluronic acid gel	192	Postoperative Adhesion of Uterus
Efficiency of INTERCOAT (Oxiplex/AP Gel) in Preventing Intrauterine Adhesion Formation in Hysteroscopic Surgery	NCT01637974	Crosslinked hyaluronic acid gel	130	Asherman Syndrome; Endometrial Polyp; Uterine Myoma
Efficiency of Intercoat (Oxiplex/AP Gel)in Decreasing Intrauterine Adhesions	NCT01377779	Crosslinked hyaluronic acid gel	60	Asherman Syndrome
Hyaluronic Acid and Uterine Synechiae	NCT02248376	Crosslinked hyaluronic acid gel	364	Uterine Synechiae After Scraping for Natural Miscarriage
Prevention of Intrauterine Adhesions After Hysteroscopic Metroplasty With Autocross-linked Hyaluronic Acid Gel	NCT02404454	Crosslinked hyaluronic acid gel	50	Intrauterine Adhesions; Septate Uterus
Safety Study of Use of Hyaluronic Acid Gel To Prevent Intrauterine Adhesions In Hysteroscopic Surgery	NCT01464528	Crosslinked hyaluronic acid gel	10	Tissue Adhesions
Effectiveness of Hyaluronic Acid Gel in the Prevention of Intrauterine Adhesions After Second Trimester Abortion	NCT02868437	Crosslinked hyaluronic acid gel	60	Placenta Retained; Uterine Diseases
The Efficacy of A New Crosslinked Hyaluronan Gel in Prevention of Intrauterine Adhesion	NCT03353909	Crosslinked hyaluronic acid gel	300	Intrauterine Adhesion
Seprafilm® Adhesion Barrier and Cesarean Delivery	NCT00565643	modified sodium hyaluronic acid and carboxymethylcellulose	753	AdhesionsCesarean SectionDelivery, Obstetric

Estrogen has been shown to stimulate endometrial repair, prevent adhesion recurrence, and improve menstrual status. However, the clinical application of estrogen therapy is only empirical, and there is no unified application specification for dosage. It is hypothesized that if large doses of estrogen are given immediately following surgery, the residual endometrium in the uterine cavity will overgrow, preventing new damage.^120^ Aspirin, sildenafil citrate, nitroglycerin, and other drugs have been shown in studies to increase endometrial blood perfusion and improve pregnancy outcomes. It is hoped that it will be used in clinical trials.^121^ Platelet-rich plasma therapy has been used by several researchers. Platelet-rich plasma, which contains various growth factors and platelets, is the basis for these therapies. G-CSF has been used in some studies to demonstrate contradictory efficacy.^122^ According to the findings of Zadehmodarres’ pilot study, platelet-rich plasma promotes endometrial growth in patients with thin endometrium and pregnancy in infertile patients.^123^ Dogras et al. demonstrated that platelet-rich plasma improved endometrial thickness and pregnancy outcomes during fresh and frozen-thawed embryo transfer in women with refractory thin endometrium of various etiologies.^124^ Cheng et al. demonstrated that extracorporeal shock wave therapy can improve its efficacy by collaborating with platelet-rich plasma.^122^ These measures can be used in conjunction with uterine tissue engineering, but severe injuries such as partial or subtotal hysterectomy still necessitate more sophisticated tissue engineering systems.

### Future of uterine tissue engineering

With an increasing number of studies being focused on the clinical trial stage, many better clinical products are expected to be developed to treat various obstetric and gynecological conditions such as endometrial injury, uterine defect, and thin endometrium. In short, HA products and derivatives may be available on the market (Tables 2 and 3). The use of cell products such as proteins, cytokines, and exosomes to improve their biological activities may be seen in the next generation of uterine tissue engineering. New materials can help cells survive, differentiate, and function better. The investigation of large acellular scaffolds has yielded numerous promising results for complex-shaped defects. Modifying scaffold properties will aid in the development of multifaceted materials that 1) subtly control various properties to regulate physical and chemical properties; 2) regulate immune and regeneration signaling pathways; and 3) produce positive clinical results. Our ultimate goal was to replace all of the missing tissue without causing any harm until the uterus was completely replaced. However, many challenges remain, such as 1) local inflammation of materials; 2) incomplete differentiation of stem cells; and 3) transplanted tissue death. A synergy between material science and cell technology can be achieved as this field advances, resulting in an effective and important platform for future preclinical and clinical applications in obstetrics and gynecology.

### Conclusion

Developing uterine tissue engineering into a clinically viable treatment is becoming a more realistic goal. The simultaneous interpretation of bioactive cells and biomaterial scaffolds may be a strategy for improving human uterine injury. Stem cell technology also promotes the development of this field. The current state of knowledge on cell sources, structures, biological factors, and biomaterial scaffolds for creating functionally engineered uterine tissues is discussed in this review. Further research should be conducted to prove the structure’s full maturity, functional integration, longevity, and scalability. We may be able to rebuild the uterus by coordinating the efforts of various researchers involved in this field to achieve functional goals.
